# Inhibition of Akt sensitises neuroblastoma cells to gold(III) porphyrin 1a, a novel antitumour drug induced apoptosis and growth inhibition

**DOI:** 10.1038/sj.bjc.6605147

**Published:** 2009-06-23

**Authors:** W Li, Y Xie, R W-Y Sun, Q Liu, J Young, W-Y Yu, C-M Che, P K Tam, Y Ren

**Affiliations:** 1 Department of Surgery, University of Hong Kong, Pokfulam Road, Hong Kong, PR China; 2 Laboratory in Department of Surgery, The First Affiliated Hospital, Guangzhou Medical College, Guangzhou, Guangdong, PR China; 3 Department of Chemistry, University of Hong Kong, Pokfulam Road, Hong Kong, PR China; 4 Department of Cell Biology and Neuroscience, Rutgers University, NJ 08854, USA

**Keywords:** Akt, apoptosis, caspases, gold(III) porphyrin 1a, neuroblastoma

## Abstract

**Background:**

Gold(III) porphyrin 1a is a new class of anticancer drug, which inhibits cell proliferation of wide range of human cancer cell lines and induces apoptosis in human nasopharyngeal carcinoma cells. However, the underlying signalling mechanism by which gold(III) porphyrin 1a modifies the intracellular apoptosis pathways in tumour cells has not been explained in detail in neuroblastoma cells.

**Methods:**

Cell proliferation and apoptosis were determined by measuring 3-(4,5-dimethylthiazol-2-yl)-2,5-diphenyltetrazolium bromide (MTT) and Annexin V binding, respectively. Western blot assay was used to detect proteins involved in apoptotic and Akt pathways. *In vivo* tumour growth was assessed by inoculating tumour cells to nude mice subcutaneously, and gold(III) porphyrin 1a was administrated intravenously.

**Results:**

This study assessed the antitumour effect and mechanism of gold(III) porphyrin 1a on neuroblastoma *in vitro* and *in vivo*. Gold(III) porphyrin 1a displayed a growth inhibition and induction of apoptosis in neuroblastoma cells effectively *in vitro*, which was accompanied with release of cytochrome *c* and Smac/DIABLO and caspases activation. Further studies indicated that gold(III) porphyrin 1a inhibited X-linked inhibitor of apoptosis (XIAP). However, we found that gold(III) porphyrin 1a can induce a survival signal, Akt activation within minutes and could last for at least 24 h. To further confirm association between activation of Akt and the effectiveness of gold(III) porphyrin 1a, neuroblastoma cells were treated with API-2, an Akt-specific inhibitor. API-2 sensitised cells to gold(III) porphyrin 1a-induced apoptosis and growth inhibition.

**Conclusion:**

These results suggested that Akt may be considered as a molecular ‘brake’ that neuroblastoma cells rely on to slow down gold(III) porphyrin 1a-induced apoptosis and antiproliferation. Gold(III) porphyrin 1a is a mitochondrial apoptotic stimulus but also activates Akt, suggesting an involvement of Akt in mediating the effectiveness to growth inhibition and apoptosis by gold(III) porphyrin 1a and that inhibition of Akt can enhance the anticancer activity of gold(III) porphyrin 1a in neuroblastoma.

Neuroblastoma is one of the most common malignant tumours in children, and is responsible for about 15% of all paediatric cancer deaths. (Maris and Matthay, 1999) Spontaneous regressions and differentiation *in vivo* are common in infants and in early-stage tumours, whereas children who are over 1 year of age at clinical presentation often have metastatic disease that fails to respond to current therapies, including intensive chemotherapy, irradiation therapy, and surgery. (Matthay *et al*, 1999; Weinstein *et al*, 2003) The underlying biological mechanism has not been fully elucidated. Despite recent advances in the management of the disease by conventional chemotherapy, the development of new therapeutic strategies is critically important.

Apoptosis is mediated by two central pathways: the extrinsic (or death receptor) and the intrinsic (or mitochondrial) pathways (Danial and Korsmeyer, 2004). The intrinsic pathway is initiated through either the disruption of the mitochondrial membrane potential directly or indirectly through upstream effector molecules. This disruption leads to the release of cytochrome *c* and the formation of the apoptosome complex of cytochrome *c*/caspase 9/Apaf-1 and activation of the caspase cascade leading to apoptosis. Several endogenous proteins antagonize the intrinsic pathway such as X-linked inhibitor of apoptosis (XIAP). X-linked inhibitor of apoptosis binds to and inhibits caspase-9, 3, and 7 (Deveraux *et al*, 1998). The release of Smac/DIABLO from mitochondrial to the cytosol opposes the inhibition of caspases by XIAP (Du *et al*, 2000). This relief of inhibition is mediated by binding Smac/DIABLO to XIAP, displacing the caspases. The role of Smac/DIABLO emphasizes that although release of cytochrome *c* is necessary, it is not sufficient in many situations to bring about cell death (Potts *et al*, 2003). Inactivation of the ‘cell death brakes’ by these other apoptogens is also often necessary for apoptosis to occur (Crow *et al*, 2004).

The phosphatidylinositol 3′-kinase (PI3K)–Akt signalling pathway regulates fundamental cellular functions such as transcription, translation, proliferation, growth, and survival (Osaki *et al*, 2004). It has been shown that Akt is capable of interfering with both the intrinsic (mitochondrial) and extrinsic (receptor mediated) apoptotic pathways (Nicholson and Anderson, 2002). Inhibiting Akt signalling in tumour cells led to selective induction of apoptosis in tumour cells expressing activated Akt. In contrast, a minimal effect was observed in normal or tumour cells expressing low levels of active Akt (Jetzt *et al*, 2003). These studies state the importance of Akt activation in tumour cell survival. Recent studies have shown that the activation of Akt also contributes to both chemotherapeutic resistance and radiation resistance in non-small cell lung cancer and other carcinoma (Brognard *et al*, 2001; Clark *et al*, 2002). However, the importance of Akt in promoting therapeutic resistance in neuroblastoma cells has not been established.

Gold(III) porphyrin 1a is a novel antitumor chemotherapeutic reagent (Che *et al*, 2003). Preliminary data showed that gold(III) porphyrin 1a inhibited cell proliferation of wide range of human cancer cell lines, including human cervical epithelioid cancer cells, leukaemia, hepatocellular, and nasopharyngeal carcinoma cells (Che *et al*, 2003; Wang *et al*, 2005) *in vitro*, suggesting that this molecule may offer novel approach to cancer therapy. Very recent data showed that gold(III) porphyrin 1a induced apoptosis in human nasopharyngeal carcinoma cells through mitochondrial death pathways related to reactive oxygen species (ROS) (Wang *et al*, 2005, Wang *et al*, 2006). However, the underlying signalling mechanism by which gold(III) porphyrin 1a modifies the intracellular apoptosis pathways in tumour cells has not been explained in detail in neuroblastoma cells. Thus, in this study, we reported that gold(III) porphyrin 1a induced neuroblastoma cell apoptosis and growth inhibition both *in vitro* and *in vivo*. We showed that gold(III) porphyrin 1a-induced apoptosis was through the activation of caspases and mitochondria pathway. Most interestingly, gold(III) porphyrin 1a lead to the activation of Akt before the onset of apoptosis, suggesting that early activation of Akt by gold(III) porphyrin 1a may act as a molecular brake on gold(III) porphyrin 1a-induced apoptosis and thus may serve as new mechanism of therapeutic resistance. Inhibition of Akt activity might increase therapeutic efficacy of gold(III) porphyrin 1a in neuroblastoma.

## Materials and methods

### Reagents

Gold(III) porphyrin 1a was synthesised and purified by a method described previously (Che *et al*, 2003). Antibodies for phosphorylated Akt (p-Akt), Akt, caspase-3, caspase-9, PARP, and *β*-actin were from Cell Signaling (Cell Signaling Technology, Beverly, MA, USA). Chemiluminescence Western Blot detection reagents were from Perkin-Elmer (Boston, MA, USA). All other chemicals were purchased from Sigma (St Louis, MO, USA). Tissue culture plastic was purchased from Corning (Corning, NY, USA). Cell culture medium DMEM, fetal bovine serum (FBS) and antibiotics were from Mediatech Inc. (Herndon, VA, USA).

### Cell lines and culture

Neuroblastoma cell lines SK-N-AS and SK-N-SH were obtained commercially from American Type Culture Collection (ATCC, Rockville, MD, USA) and cells were cultured with DMEM medium supplemented with 10% (v/v) heat inactive FBS, 100 U ml^−1^ penicillin and 100 *μ*g ml^−1^ streptomycin in a humidified 5% CO_2_ incubator at 37°C. The human non-tumorigenic, immortalised liver cell line MIHA was maintained in Waymouth's MB 752/1 medium (Gibco BRL, Grand Island, NY, USA) supplemented with 10% FBS, 100 U ml^−1^ penicillin, 100 mg ml^−1^ streptomycin, 50 mM dexamethasone and 20 mU ml^−1^ insulin (Boehringer Mannheim, Indianapolis, IN, USA).

### Apoptosis assay

Apoptosis was detected by fluorescein-labelled Annexin V staining using the Annexin V-Fluos staining kit (Roche, Mannheim, Germany). Cells were treated with gold(III) porphyrin 1a at different concentrations. Cells were washed with ice-cold phosphate-buffered saline (PBS), trypsinised, and labelled with Annexin V for 15 min at room temperature in the dark. The percentage of FITC-positive cells was analysed by flow cytometry (FACScaliber, Becton Dicknison, Mountain View, CA, USA). The data were analysed using CellQuest software (Becton Dicknison).

### Proliferation assay

Cells were treated with different concentrations of gold(III) porphyrin 1a from 0.125 to 1.0 *μ*M for 48 h. Cell proliferation was determined by measuring 3-(4,5-dimethylthiazol-2-yl)-2,5-diphenyltetrazolium bromide (MTT) reduction following the protocol (Cell Proliferation kit, BD Biosciences, San Jose, CA, USA), with a microtiter plate reader (Bio-Rad Model 550, Hercules, CA, USA) at 570 nm. Optical density was measured at 570 nm. Data were obtained from three separate experiments. The percentage of cell survival was determined by comparing the absorbance value of the vehicle control.

### Isolation of cytosolic fractions

The assay was performed according to the method of (Bossy-Wetzel *et al* (1998) with minor modifications. Cells were washed in cold PBS twice and resuspended in mitochondria isolation buffer (20 mM Hepes, pH 7.5, 1.5 mM MgCl_2_, 10 mM KCl, 1 mM EDTA, 1 mM EGTA, 1 mM dithiothreitol, 0.1 mM phenylmethylsulfonyl fluoride, 10 *μ*g ml^−1^ leupeptin, aprotinin, and pepstatin containing 250 mM sucrose). Cells were homogenised in an ice-cold tissue grinder with 10 strokes. The homogenate was centrifuged at 900 **g** for 10 min. The resulting supernatant was centrifuged at 13 000 **g** for 15 min and pellet was designated as mitochondria. The supernatant was further centrifuged at 13 000 **g** to remove any other particulate material and supernatant was used as the cytosolic fraction.

### Western blot

Cells were washed with PBS, and directly lysed in lysis buffer (50 mM Tris-HCl (pH=8.0), 1% Triton X-100, 10% glycerol, 1 mM EDTA, 250 mM NaCl, 1 mM dithiothreitol, 1 mM phenylmethylsulfonyl fluoride, 2 mM sodium vanadate, 100 mM sodium fluoride, 10 *μ*g ml^−1^ aprotinin, 10 *μ*g ml^−1^ leupeptin and 10 *μ*g ml^−1^ pepstatin). Cell lysates were adjusted to equal protein concentrations (Bio-Rad Protein Assay, Hercules, CA, USA), resuspended in 2 × sample loading buffer containing 4% SDS, 20% glycerol, 120 mM Tris and bromophenol blue, and were boiled for 5 min. Protein samples were subjected to SDS-polyacrylamide gel electrophoresis. Proteins on the gel were transferred onto nitrocellulose membranes that were blocked with 5% milk in Tris-buffered saline containing 0.1% Tween 20 (TBST) for 1 h at room temperature. Afterwards, the membranes were incubated with the indicated primary antibodies overnight at 4°C. After being washed with TBST, the membranes were incubated with the appropriate secondary antibody. The immunoreactive bands were visualised with chemiluminescence.

### Tumour growth in nude mice

Six-week-old BALB/c nude mice (Laboratory Animal Unit, The University of Hong Kong) were injected with neuroblastoma cell lines SK-N-SH and SK-N-AS subcutaneously with 1 × 10^6^ cell. After 20 days, all of the nude mice developed the tumours. For treatment group (*n*=10), 10 mg kg^−1^ gold(III) porphyrin 1a dissolved in 10% ethanol was administrated intravenously on day 21 postinoculation. Control group (*n*=10) received 10% ethanol vehicle. Tumour growth in each group was monitored everyday by two-dimensional measurements of individual tumours.

## Results

### Effects of gold(III) porphyrin 1a on tumour growth *in vivo*

**Figure 1:**
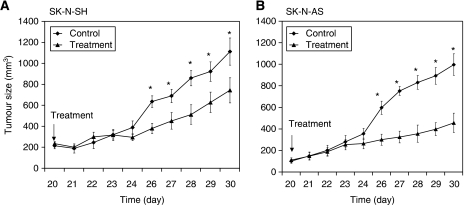
Antitumor effect of gold(III) porphyrin 1a in BALB/c-nude mice. Mice were inoculated with SK-N-SH (**A**) and SK-N-AS (**B**) subcutaneously with 1 × 10^6^ cells. Tumours developed 20 days after injection and mice were administered gold(III) porphyrin 1a every 3 days intravenously until day 21 postinoculation. Control group received vehicle alone (10% ethanol). The size of tumours in mice was monitored everyday. Results are expressed as the mean (*n*=10 per group, ^*^P<0.05).

Mice were inoculated with neuroblastoma cell lines SK-N-AS and SK-N-SH subcutaneously with 1 × 10^6^ cells. SK-N-SH cell line possesses multidrug resistance (MDR) phenotype and SK-N-AS cell line is non-MDR phenotype. (Cinatl *et al*, 1999) Mice were administered gold(III) porphyrin 1a (10.0 mg kg^−1^) every 3 days intravenously till day 21 postinoculation. Control group received vehicle alone. Tumour growth in each group was monitored everyday by two-dimensional measurements of individual tumours. In control group, tumours in mice inoculated with SK-N-AS and SK-N-SH grew very progressively. However, treatment with gold(III) porphyrin 1a resulted in strong and significant decreases in tumour size both in mice inoculated with SK-N-AS and SK-N-SH (Figure 1A and B), suggesting that gold(III) porphyrin 1a not only inhibited non-MDR neuroblastoma growth (SK-N-AS) but also was effective for MDR phenotype neuroblastoma (SK-N-SH). In addition, non-MDR cell line SK-N-AS responded significantly better than the MDR cell line SK-N-SH (*P*<0.05).

### Antiproliferative effects and apoptosis induction of gold (III) porphyrin 1a

**Figure 2:**
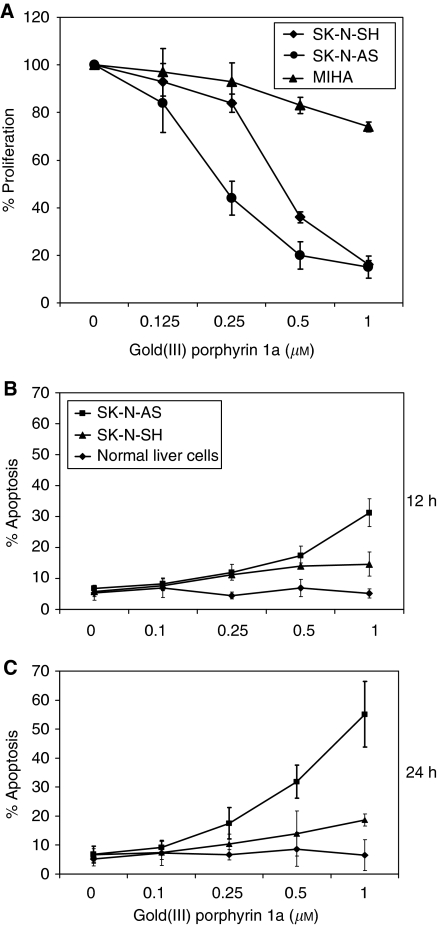
Proliferative and apoptotic effects of gold(III) porphyrin 1a on neuroblastoma cells. (**A**) SK-N-SH, SK-N-AS and MIHA (normal liver cell line) cells were cultured with gold(III) porphyrin 1a for 48 h in 96-well plate. The proliferation was evaluated by MTT assay. Each treatment group contained eight replicates. Data expressed as mean±s.d. and similar results were obtained from independent experiments. (**B** and **C**) SK-N-SH, SK-N-AS, and MIHA cells were cultured with gold(III) porphyrin 1a for 12 h (**B**) and 24 h (**C**). Apoptosis of gold(III) porphyrin 1a on neuroblastoma cells was evaluated by Annexin V staining. Data expressed as mean±s.d. and similar results were obtained from independent experiments.

Given these findings *in vivo*, we further analysed how gold(III) porphyrin 1a affects tumour growth *in vitro.* The effects of gold(III) porphyrin 1a on cell proliferation were assessed in the human neuroblastoma cell lines SK-N-SH and SK-N-AS. Gold(III) porphyrin 1a-induced growth inhibitory effects occurred in SK-N-SH and SK-N-AS with IC_50_ values 0.2 and 0.4 *μ*M, respectively. However, under the same conditions, the effect of gold(III) porphyrin 1a on normal live cell (MIHA) was lowered when compared with SK-N-SH and SK-N-AS (Figure 2A). SK-N-SH cell line has MDR phenotype and the IC_50_ values for cisplatin, a common first-line chemotherapeutic drug in clinical setting, exceeded 40 *μ*M for SK-N-SH and was 10.5 *μ*M for SK-N-AS (Table 1). SK-N-SH is resistant to cisplatin treatment; however, the sensitivity to gold(III) porphyrin 1a is 100 times more than cisplatin.

To understand whether gold(III) porphyrin 1a has the effect on tumour cell apoptosis, cells were treated with gold(III) porphyrin 1a for 12 or 24 h and apoptosis was assessed by Annexin V binding assay. Gold(III) porphyrin 1a induced apoptosis in SK-N-AS and SK-N-SH but had no effect in normal live cells. Similarly, gold(III) porphyrin 1a induced more apoptosis at 24 h compared with 12 h (Figure 2B and C). Therefore, gold(III) porphyrin 1a-elicited neuroblastoma cell apoptosis was in time- and dose-dependent manners.

### Gold(III) porphyrin 1a induced release of cytochrome *c* and Smac/Diablo

**Figure 3:**
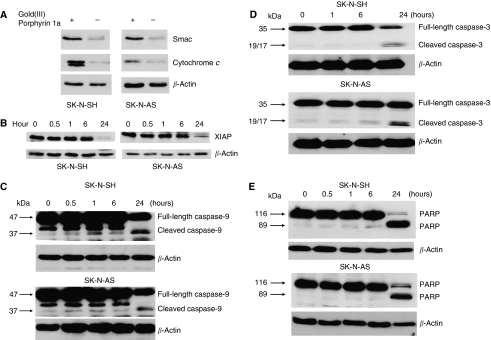
Characterisation of gold(III) porphyrin 1a-mediated apoptosis in neuroblastoma cells. (**A**) Smac and cytochrome *c* release in neuroblastoma cells treated with gold(III) porphyrin 1a. SK-N-SH and SK-N-AS were treated with gold(III) porphyrin 1a (0.5 *μ*M) for 8 h and Smac and cytochrome *c* in cytosol were detected by western blot analysis. SK-N-SH and SK-N-AS cells were also treated with gold (III) porphyrin 1a (0.5 *μ*M) for different periods of time. Western blotting was applied to analyse the expression of full-length XIAP (53 kDa) (**B**), full-length (47 kDa) and cleaved caspase-9 (37/35 kDa) (**C**), full-length (35 kDa) and cleaved caspase-3 (19/17 kDa) (**D**), and full-length (116 kDa) and cleaved PARP (89 kDa) (**E**).

We further explored the mechanism of gold(III) porphyrin 1a-induced apoptosis of neuroblastoma cells. Apoptotic stimuli can damage mitochondrial, resulting in cytochrome *c* and mitochondrial protein Smac/Diablo release into the cytosol. Having previously reported the release of cytochrome *c* with gold(III) porphyrin 1a treatment in human nasopharyngeal carcinoma cells (Wang *et al*, 2005), we next assessed whether Smac/Diablo was also released. SK-N-SH and SK-N-AS were treated with gold(III) porphyrin 1a (0.5 *μ*M) for 8 h and cytosols were separated. As showed in Figure 3A, Smac/Diablo was released to cytosol in a pattern similar to cytochrome *c*.

### Gold(III) porphyrin 1a treatment resulted in downregulation and cleavage of XIAP

Smac/Diablo is known to exert its proapoptotic effect by inhibition of XIAP. X-linked inhibitor of apoptosis is a member of intracellular antiapoptotic proteins that confers protection form death-inducing stimuli by directly blocking the activation of caspases (Deveraux *et al*, 1999). To determine the effect of XIAP in gold(III) porphyrin 1a-induced apoptotic execution, cells were treated with gold(III) porphyrin 1a (0.5 *μ*M) for different periods of time. Expression of full-length XIAP (53 kDa) in both SK-N-SH and SK-N-AS was markedly inhibited by gold(III) porphyrin 1a at time 24 h (Figure 3B). The data also showed that the time of Smac/Diablo release preceded that of XIAP cleavage (Figure 3A and B).

### Effect of gold(III) porphyrin 1a on the activation of caspases

Activation of caspases during apoptosis results in the cleavage and activation/inactivation of a range of critical cellular substrates, including the DNA repair enzyme PARP. To determine which caspases were involved in gold(III) porphyrin 1a-induced apoptosis, the expressions of activated caspases were detected by western blotting. As shown in Figure 3C–E, in control cells, caspase-9 and -3 and PARP were all present as uncleaved forms. After the treatment with gold(III) porphyrin 1a for 24 h, the cleavage of caspases and PARP to catalytically active fragments was clearly detected, suggesting the activation of these caspases.

### Gold(III) porphyrin 1a induced phosphorylation of Akt

**Figure 4:**
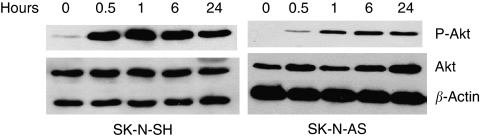
Effect of gold(III) porphyrin 1a on induction of Akt activity. SK-N-SH and SK-N-AS cells were treated with gold(III) porphyrin 1a (0.5 *μ*M) for different periods of time. Akt phosphorylation and total Akt expression was analysed by western blotting.

As Akt activation has been implicated in the promotion of tumour cell proliferation and some antitumour drugs can inhibit tumour growth by inactivating Akt, we next examined whether gold(III) porphyrin 1a could affect Akt. SK-N-SH and SK-N-AS cells were treated with gold(III) porphyrin 1a (0.5 *μ*M) for different periods of time. Interestingly, we found that Akt activation was not inhibited by gold(III) porphyrin 1a. By contrast, gold(III) porphyrin 1a treatment resulted in Akt activation by phosphorylation of Akt at Ser473 beginning by 30 min in both neuroblastoma cells SK-N-AS and SK-N-SH (Figure 4), suggesting an early induction of Akt before the onset of apoptosis. Furthermore, the activation of Akt can last for at least 24 h.

### Effect of inhibition of Akt activity on the sensitivity of cells to gold(III) porphyrin 1a treatment

**Figure 5:**
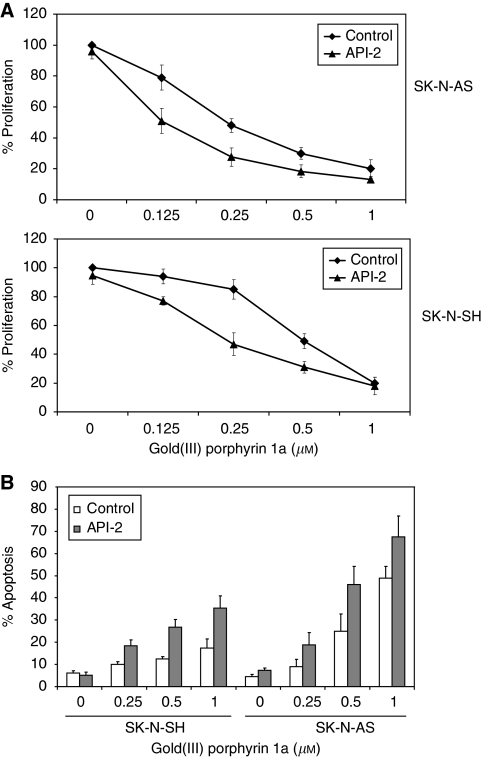
Inhibition of Akt activity sensitises cells to gold(III) porphyrin 1a-induced proliferative inhibition and apoptosis. (**A**) SK-N-SH and SK-N-AS cells were pretreated with or without API-2 (1 *μ*M) for 2 h and then cultured with gold(III) porphyrin 1a for 48 h. Proliferation was assessed by MTT assay. Each treatment group contained eight replicates. Data were expressed as mean±s.d.; similar results were obtained from repeated experiments. (**B**) SK-N-SH and SK-N-AS cells were pretreated with or without API-2 (1 *μ*M) for 2 h and then cultured with gold(III) porphyrin 1a for 24 h. Apoptosis was assessed by Annexin V binding assay. Data were expressed as mean±s.d.; similar results were obtained from duplicate experiments.

Because activation of Akt can protect cells from apoptosis and our data showed gold(III) porphyrin 1a-induced early activation of Akt, we therefore reasoned that whether inhibition of Akt activation might sensitise cells to gold(III) porphyrin-induced apoptosis. Akt/protein kinase B signalling inhibitor (API-2), a cell-permeable tricyclic nucleoside, selectively inhibited cellular phosphorylation of Akt but not activation of PI3K, PDK1, PKC, PKA, ERK1/2, or c-Jun NH2-terminal kinase (Yang *et al*, 2004). Neuroblastoma cells SK-N-AS and SK-N-SH were pretreated with or without API-2 (1.0 *μ*M) for 2 h and then treated with gold(III) porphyrin 1a for 24 h. API-2 at 1.0 *μ*M can inhibit Akt activation (data not shown) but did not affect cell growth and apoptosis (Figure 5). Treatment with API-2 enhanced tumour cell sensitivity to gold(III) porphyrin 1a-induced growth inhibition in a dose-dependent manner (Figure 5A). The IC_50_ values for gold(III) porphyrin 1a in SK-N-AS and SK-N-SH treated with API-2 were 0.1 and 0.2 *μ*M compared with 0.2 and 0.4 *μ*M in cells treated with gold(III) porphyrin 1a alone (Figure 5A). To assess how inhibition of Akt activation would affect gold(III) porphyrin 1a-induced apoptosis, cells were treated with gold(III) porphyrin alone or the combinations of API-2 (1.0 *μ*M) and gold(III) porphyrin 1a for 24 h. When treatment was combined with API-2, gold(III) porphyrin 1a resulted in dose-dependent apoptosis induction that was greater than that observed at the same concentration of gold(III) porphyrin 1a without API-2 treatment (Figure 5B), which is consistent with the antiproliferative assay results.

## Discussion

Gold(III) porphyrin 1a, a chemically synthetic compound with a simple chemical structure, is a novel class of antitumour drug (Che *et al*, 2003). It displayed antiproliferative activity in several types of human cancer cells (Wang *et al*, 2005, Wang *et al*, 2006; Lum *et al*, 2006). However, the precise target and mechanism of action of gold(III) porphyrin 1a on tumour cells remain largely unknown.

In this study, we showed that gold(III) porphyrin 1a induced apoptosis and growth inhibition in neuroblastoma cells, including MDR cells *in vivo* and *in vitro*. Importantly, there is no cytotoxicity in normal liver cells and peripheral blood mononuclear cells (data not shown). This *in vitro* result was confirmed by that of the *in vivo* study. Gold(III) porphyrin 1a treatment was active against neuroblastoma xenografts and significantly inhibited tumour growth. These data show that the antitumor activity exerted by gold(III) porphyrin 1a is effective, *in vivo*, in treating cancer.

Numerous stimuli and stresses are able to stimulate the release of apoptogenic factors from the mitochondrial intermembrane space, leading to the programmed demise of the cell. To understand the mechanism by which gold(III) porphyrin 1a induces apoptosis of tumour cells, we have examined the changes in the expression and localisation of several apoptosis-related proteins in neuroblastoma cells exposed to gold(III) porphyrin 1a. The western blot analysis revealed that gold(III) porphyrin 1a induced the release of cytochrome *c* and Smac/Diablo, activation of caspase-9 and -3, and PARP cleavage, indicating that gold(III) porphyrin 1a-induced apoptosis might be primarily mediated by the mitochondrial (intrinsic) apoptosis pathway. We further explored the expression of XIAP in neuroblastoma cell lines. We found that SK-N-SH and SK-N-AS expressed high levels of XIAP. These findings correlated with previous studies showing that IAPs are highly expressed in many tumours and contribute to the resistance of cancers to apoptosis (Deveraux *et al*, 1998; Salvesen and Duckett, 2002; Kamsteeg *et al*, 2003). Treatment of the neuroblastoma cells with gold(III) porphyrin 1a inhibited the expression of XIAP. It has been shown that the cleavage of XIAP may be one of the mechanisms by which cell death programs circumvent the antiapoptotic barrier posed by XIAP (Deveraux *et al*, 1999). This may explain our findings of caspase-9 activation after gold(III) porphyrin 1a treatment.

Recent study showed that the majority of cytochrome *c* release was abolished in the Smac-KO cells, suggesting that Smac plays an important role in promoting cytochrome *c* release and full execution of NSAID-induced apoptosis (Bank *et al*, 2008). Studies by other groups also showed that the release of different mitochondrial apoptogenic proteins is coordinated and occurs through different time courses (Munoz-Pinedo *et al*, 2006). Our data showed for the first time that gold(III) porphyrin 1a not only enhanced cytochrome *c* release but also promoted Smac release. Smac release may be critical for its ability to promote caspase activation and cytochrome *c* release during gold(III) porphyrin 1a-induced apoptosis. The release of Smac and cytochrome *c* may have synergetic role on apoptosis by the activation of caspase-9 and inhibition of XIAP. Further study is needed to explore the requirement of Smac for full execution of gold(III) porphyrin 1a-induced apoptosis in neuroblastoma cells.

It is well documented that Akt can promote cellular survival. Akt is activated in response to many different growth factors (Datta *et al*, 1999). Previous studies also showed that Akt plays important roles in cell survival when cells are treated with different apoptotic stimuli such as growth factor withdrawal, UV irradiation, and etoposide (Widmann *et al*, 1998). Akt protects cells from apoptosis by phosphorylating and inactivating several key apoptotic molecules: Bad, procaspase-9, and FKHR1 (Pommier *et al*, 2004). Our results showed for the first time that gold(III) porphyrin 1a not only enhanced neuroblastoma apoptosis but also activated Akt, the survival signal. Inhibition of Akt activation sensitised neuroblastoma cells to the effects of antiproliferation and apoptosis by gold(III) porphyrin 1a. The antiproliferation and apoptosis induced by gold(III) porphyrin 1a on cells could be enhanced by the inhibition of Akt activation pharmacologically with specific Akt inhibitor, suggesting that Akt activation might play a role in the protection against the effects of apoptosis and growth inhibition of gold(III) porphyrin 1a in neuroblastoma. To our knowledge, these studies are the first to show that Akt is a survival factor for neuroblastoma cells under conditions of with administration of gold(III) porphyrin 1a, which are similar to the previous studies that showed that the Akt activity was induced by chemotherapy, doxorubicin, trastuzumab, or tamoxifen (Clark *et al*, 2002). This phenomenon was also observed by Tang *et al* (2001), who showed that Akt was activated in response to apoptotic stimuli: staurosporine and etoposide.

What is the mechanistic connection between gold(III) porphyrin 1a and Akt induction? It has been shown that gold(III) porphyrin 1a can generate ROS (Wang *et al*, 2005) which may contribute to Akt activation in a similar way to hydrogen peroxide as some apoptotic stimuli such as doxorubicin-generated ROS and UV B irradiation-induced DNA damage can activate Akt (Ushio-Fukai *et al*, 1999; Van der Kaay *et al*, 1999; Wang *et al*, 2000). However, the activation of Akt by hydrogen peroxide occurred within minutes and was transient, whereas our study showed that gold(III) porphyrin 1a-induced activation of Akt can last for at least 24 h and was not transient. This result suggests that other mechanisms may be involved to alter Akt levels in neuroblastoma cells in response to gold(III) porphyrin 1a. Other reports have showed that Akt can also be activated by other factors such as heat-shock proteins (Konishi *et al*, 1997; Rane *et al*, 2003; Solit *et al*, 2003) or by other cellular stress such as hypoxia (Alvarez-Tejado *et al*, 2001). Therefore, further studies are needed to identify the mechanisms of Akt activation induced by gold(III) porphyrin 1a.

Akt activation contributes to chemotherapeutic resistance and radiation resistance in breast, lung, prostate and ovarian carcinomas, leukaemia and melanoma (Krasilnikov *et al*, 1999; Wellbrock *et al*, 1999; Brognard *et al*, 2001; Chen *et al*, 2001; Stassi *et al*, 2005). It has been reported that a growth/survival factor-stimulated mechanism leading to chemoresistance in neuroblastoma is mediated by the PI3K/Akt signalling pathway (Li and Thiele, 2007). These findings raise the possibility of a new therapeutic approach in neuroblastoma that would target Akt to enhance the efficacy of chemotherapeutics. Combination regimens of gold(III) porphyrin 1a with an Akt inhibitor might have even great efficacy in MDR neuroblastoma cells. Several small-molecule inhibitors of the PI3K/Akt pathway are in active preclinical development (Powis *et al*, 2006). Specific Akt inhibitors such as API-2, a cell-permeable tricyclic nucleoside that selectively inhibits cellular pAkt by targeting the Akt effector molecule, do so without inhibition of activation of the upstream PI3K and PDK1 or downstream molecules (Yang *et al*, 2004). Although neuroblastoma SK-N-SH cell line assessed was less sensitive to gold(III) porphyrin 1a compared with SK-N-AS *in vitro* and *in vivo*, inhibition of Akt activation enhanced gold(III) porphyrin 1a response in these cells. Thus, p-Akt and its upstream activating pathway, PI3K, may have a critical role in conferring resistance to apoptosis induction by gold(III) porphyrin 1a. As Akt is vital for endothelial cell function and vascular integrity, its inhibition might also potentiate the antitumour effects of gold(III) porphyrin 1a (Somanath *et al*, 2006).

Taken together, our data emphasise that gold(III) porphyrin 1a is a mitochondrial apoptotic stimulus and reveal the potential importance of Akt as a therapeutic target in neuroblastoma. Akt may be considered as a molecular ‘brake’ that neuroblastoma cells rely on to slow down gold(III) porphyrin 1a-induced apoptosis and antiproliferation. It is possible that the activation of Akt inhibits the desired antitumor effects of gold(III) porphyrin 1a, particularly in the cells in which Akt is largely amplified. Inhibition of Akt activity by specific inhibitor can remove the apoptotic ‘brake’ and sensitise the neuroblastoma cells to gold(III) porphyrin 1a treatment.

## Figures and Tables

**Table 1:** Cytotoxicity (IC_50_) of Gold(III) porphyrin on neuroblastoma cell lines

	**IC_50_ (*μ*M)**	
**Cell lines**	**Gold(III) porphyrin 1a**	**Cisplatin**
SK-N-AS (non-MDR phenotype)	0.2	10.5
SK-N-SH (MDR phenotype)	0.4	>40.0
Normal liver cells	1.75	38.5

MDR=multidrug resistance.
